# Qualité des prescriptions antibiotiques et déterminant de leur conformité dans les services de gynécologie du Centre Hospitalier Universitaire Souro Sanou de Bobo-Dioulasso

**DOI:** 10.11604/pamj.2026.53.135.50786

**Published:** 2026-03-23

**Authors:** Arsène Quddus Zouré, Wendin-Manegdé Félicité Nana, Relwendé Louis Arnaud Ouédraogo

**Affiliations:** 1 Université Nazi Boni/Centre d'Excellence Africain en Innovations Biotechnologiques pour l'Elimination des Maladies à Transmission Vectorielle, Ouagadougou, Burkina Faso,; 2 Institut de Recherche en Science de la Santé, Ouagadougou, Burkina Faso

**Keywords:** Antibiotique, résistance aux antimicrobiens, audit clinique, gynécologie, Burkina Faso, Antibiotics, antimicrobial resistance, clinical audit, gynecology, Burkina Faso

## Abstract

**Introduction:**

l'usage inapproprié des antibiotiques constitue un facteur d'émergence des résistances antimicrobiennes et représente un problème majeur de santé publique. Pour y faire face, le Burkina Faso a élaboré un guide pratique pour la bonne prescription des antibiotiques. Quatre ans après sa diffusion, aucune évaluation formelle de son application n'a été retrouvée, justifiant l’intérêt de cette étude.

**Méthodes:**

une étude transversale a été conduite au département de gynécologie-obstétrique et de médecine de la reproduction du Centre Hospitalier Universitaire Souro Sanou du Burkina Faso du 1^er^ juillet au 31 décembre 2022. Elle a inclus toutes les patientes ayant bénéficié d'une antibiothérapie. L'évaluation de la conformité des prescriptions a été réalisée à l'aide d'une check-list élaborée sur la base du guide national.

**Résultats:**

au total, 340 patientes ont été incluses avec un âge médian de 26 ans. Les antibiotiques les plus prescrits étaient les bêta-lactamines (principalement la ceftriaxone, 24,4%) suivis des nitromidazolés (21,4%). Le taux de conformité globale aux recommandations était de 12,4%. Les principales non-conformités concernaient la durée du traitement (87,7%), la posologie (67,7%), le choix de la molécule (60,3%) et l'indication (45,7%). L'hospitalisation ressortait comme le facteur de risque majeur de non-conformité.

**Conclusion:**

le bon usage des antibiotiques demeure un défi majeur particulièrement dans les pays en voie de développement comme le Burkina Faso. Le taux élevé de non-conformité souligne la nécessité d'actions urgentes afin de renforcer la vulgarisation du guide, d'assurer une formation continue des prescripteurs et de mettre en place des audits réguliers.

## Abstract (en)

**Introduction:**

inappropriate use of antibiotics is a major driver of antimicrobial resistance and represents a significant public health concern. To address this issue, Burkina Faso developed a practical guide for appropriate antibiotic prescribing. Four years after its dissemination, no formal evaluation of its implementation had been reported, which justified the present study.

**Methods:**

we performed a cross-sectional study in the Department of Gynecology, Obstetrics and Reproductive Medicine at the Souro Sanou University Hospital Center in Burkina Faso from July 1 to December 31, 2022. All female patients who received antibiotic therapy were included. Adherence of antibiotic prescriptions to the national guideline was assessed using a checklist developed on the basis of the national guideline.

**Results:**

a total of 340 patients were included, with a median age of 26 years. The most frequently prescribed antibiotics were beta-lactams (mainly ceftriaxone, 24.4%), followed by nitroimidazoles (21.4%). Overall compliance rate with the recommendations was 12.4%. The main non-compliances concerned treatment duration (87.7%), dosage (67.7%), choice of molecule (60.3%), and indication (45.7%). Hospitalization emerged as the main risk factor for non-compliance.

**Conclusion:**

appropriate antibiotic use remains a major challenge, particularly in developing countries such as Burkina Faso. The high rate of non-compliance highlights the urgent need to strengthen guideline dissemination, ensure continuous training of prescribers, and implement regular clinical audits.

## Introduction

L'introduction des antibiotiques dans la pratique médicale dans les années 1940 a profondément révolutionné la prise en charge des maladies infectieuses [1]. Cependant celle-ci demeure aujourd'hui encore un motif fréquent de consultation dans les centres de santé en Afrique. Au Burkina Faso par exemple, les infections respiratoires aiguës représentaient en 2024 le deuxième motif de consultation après le paludisme [2]. Cette situation explique que les antibiotiques constituent la 3^e^ classe de médicaments la plus utilisée dans le monde en milieu hospitalier [3]. Toutefois, de nombreux travaux montrent que leur utilisation reste souvent inappropriée. En France, 53% des prescriptions d'antibiotiques actifs sur les staphylocoques résistants à la méticilline (anti-SARM) étaient jugées non conformes au Centre Hospitalier Universitaire de Rouen en 2018 [4]. De même, des taux élevés de prescriptions inadaptées ont été rapportés en Tunisie (26% en antibioprophylaxie chirurgicale) [5], au Maroc (5,7% injustifiées et 15,2% non conformes aux urgences) [6], en Côte d'Ivoire (24% selon l'indication et 26% selon la posologie) [7] et au Burkina Faso, où 29,5% des prescriptions de ceftriaxone étaient inappropriées au Centre Hospitalier Universitaire Yalgado Ouédraogo en 2014 [8]. Ces mésusages favorisent l'émergence de résistances bactériennes et contribuent à une morbi-mortalité accrue [9]. On estime que les infections résistantes aux antimicrobiens (RAM) contribuent à près de 5 millions de décès par an, et pourraient être responsables de 10 millions de décès d'ici 2050, avec un coût économique global estimé à 100 milliards de dollars [10,11].

Face à ce défi de santé publique, le mot d'ordre est désormais celui du «juste usage des antibiotiques» [12]. Au Burkina Faso, cette volonté s'est traduite par l'adoption en 2017 d'un plan d'action multisectoriel de lutte contre la RAM, puis par l'élaboration en 2019 d'un guide national de bonnes prescriptions des antibiotiques. Ce document fournit des recommandations pratiques adaptées au contexte, des protocoles de traitement selon les principales infections et terrains particuliers (enfants, femmes enceintes, insuffisants rénaux, etc.), ainsi que des règles d'antibioprophylaxie. Il intègre en outre une grille d'évaluation permettant de mesurer la conformité des prescriptions. Quatre ans après sa vulgarisation, aucune évaluation formelle de son utilisation n'a toutefois été rapportée. Dans ce contexte, il apparaît essentiel de s'assurer de sa mise en œuvre effective dans les hôpitaux et d'identifier les opportunités d'amélioration. C'est dans cette optique que nous avons entrepris d'évaluer la conformité de l'usage des antibiotiques par rapport au guide au sein du département de gynécologie-obstétrique et de médecine de la reproduction (DGOMR) du Centre Hospitalier Universitaire Souro Sanou (CHUSS).

## Méthodes

**Site de l'étude:** notre étude s'est déroulée dans le département de gynécologie-obstétrique et de médecine de la reproduction (DGOMR) du Centre Hospitalier Universitaire Souro Sanou (CHUSS) de Bobo-Dioulasso au Burkina Faso.

**Type, période et population d'étude:** il s'agissait d'une étude transversale menée du 1^er^ juillet au 31 décembre 2022. Ont été incluses, toutes les patientes vues en consultation au DGOMR durant la période et ayant bénéficié d'une antibiothérapie (en hospitalisation ou en ambulatoire).

**Echantillonnage et taille d'échantillon:** nous avons procédé à un recensement de tous les dossiers. Ainsi, l'ensemble des patientes répondant aux critères d'inclusion a été pris en compte, soit un total de 340 patientes.

**Collecte des données:** les données ont été recueillies par une revue documentaire systématique à l'aide d'une grille d'analyse standardisée conçue à partir du Guide national de bonne prescription des antibiotiques (Burkina Faso, 2019). Nos sources de données étaient: les registres de consultation et d'hospitalisation; les registres du bloc opératoire; les dossiers cliniques des patientes hospitalisées.

### Définition des variables et analyse statistique

**Caractéristiques générales:** données sociodémographiques, diagnostics et modalités de prise en charge, description de l'antibiothérapie.

**La conformité de l'antibiothérapie selon:** l'indication, la molécule prescrite, la posologie, la voie d'administration, la durée du traitement et la réévaluation. Chaque critère a été codé «1» si conforme à la recommandation et «0» sinon. La conformité globale a été déclarée pour les prescriptions conformes selon l'ensemble des cinq critères.

**Les diagnostics ont été regroupés en sept catégories:** infections génitales hautes (IGH): cervicite, endométrite, myome infecté, pelvipéritonite, salpingite, pyo-salpinx ; infections génitales basses (IGB): bartholinite, vaginose, vulvovaginite, urétrite; infections urinaires/rupture prématurée des membranes (RPM); infections cutanées: abcès, érysipèle, furoncle, furonculose, mastite, ulcérations; infections du site opératoire: suppuration pariétale, textilome, endométrite (post-césarienne); autres types d'infections: bronchopneumopathie, infections neuro-méningées, sepsis; pathologies non infectieuses.

**Critères de jugement:** nous avons considéré une conformité globale reposant sur la validation de 6 critères: indication initiale à l'antibiothérapie justifiée; choix de l'antibiotique conforme selon le guide; la posologie est conforme; voie d'administration correcte; la durée totale de traitement adaptée; réévaluation faite selon la périodicité définie.

### Analyse statistique

Les données ont été saisies avec EpiData Entry v3.1 et analysées sous Stata SE v14.0. Les variables qualitatives ont été exprimées en proportions, et les variables quantitatives en moyennes ou en médiane. Pour la recherche des facteurs associés, nous avons effectué une régression logistique univariée et multivariée avec comme variable dépendante la conformité globale de prescription des antibiotiques. Les covariables étaient composées des données sociodémographiques, du type de suivi et de prescripteur ainsi que de la durée d'hospitalisation. Pour le modèle final, une valeur de p < 20% a été considérée. Les analyses ont été réalisées sur des données complètes et le seuil de significativité statistique a été établi à p < 0,05. Les graphiques et tableaux ont été élaborés à l'aide de Microsoft Office 2021.

**Considérations éthiques:** l'autorisation du directeur général du CHUSS et celle du chef du DGOMR ont été obtenues avant le début de l'étude. Le respect des principes éthiques et déontologiques a été garanti, notamment l'anonymat des patientes et des prescripteurs ainsi que la confidentialité des données.

## Résultats

### Caractéristiques générales

**Données sociodémographiques:** au total, 340 patientes ont été incluses. La médiane des âges observée était de 26 ans avec un intervalle interquartile de [21 ans, 32 ans]. Plus de la moitié résidaient en milieu urbain (58,8 %) et environ un tiers n'étaient pas scolarisées (32,4%). Parmi les patientes incluses, 70% ont été hospitalisées et 34,7% ont bénéficié d'une intervention chirurgicale (Tableau 1).

**Tableau 1:** répartition des données sociodémographiques, diagnostics et modalités de prise en charge

Caractéristiques sociodémographiques	Effectifs	Proportion (%)
**Résidence**		
Urbaine	200	58,82
Rurale	140	41,18
**Type de suivi**		
Ambulatoire	102	30
Hospitalisation	238	70
**Diagnostics**		
IGH	47	13,82
IGB	37	10,88
Infections cutanées	24	7,06
Infection site opératoire	14	4,12
Infections urinaires/RPM	19	5,59
Autres infections	13	3,82
Pathologies non infectieuses	186	54,71
**Profil prescripteurs**		
Médecin spécialiste	88	25,88
Médecin en spécialisation	220	64,71
Étudiant en 7^e^ année médecine	30	8,82
Sage-femme d'Etat/maïeuticien d'Etat	02	0,59
**Total**	**340**	**100**
Examens paracliniques		
Examen bactériologique	13	3,82
Numération formule sanguine	145	42,64
Absence examen	195	57,35
**Total**	**353**	**100**

IGH: infection génitale haute; IGB: infection génitale basse; RPM: rupture prématurée des membranes

**Caractéristiques de la prescription des antibiotiques:** les prescriptions étaient majoritairement effectuées par le personnel en formation (médecins en spécialisation et internes) dans 73,5 % des cas. Les bêta-lactamines représentaient la famille la plus prescrite 24,4% et la voie d'administration parentérale prédominait (57%) (Tableau 2).

**Tableau 2:** répartition des familles d'antibiotiques et du mode d'administration

Groupe pharmacologique	Effectifs	Proportion (%)
**Bêta-lactamines**		
Pénicillines	90	18,14
Céphalosporine 3^e^ génération	126	25,41
Nitro-imidazolés	106	21,37
Tétracyclines	52	10,48
Antifongiques / associations locales	49	9,88
Aminoglycosides	41	8,27
Macrolides	26	5,24
Fluoroquinolones	6	1,21
**Voies d'administration**		
Intra vaginale	39	7,86
Voie orale	175	35,28
Intra veineuse	277	55,85
Intra musculaire	5	1,01
**Total**	**496**	**100**

**Conformité des prescriptions:** dans notre étude, l'indication était conforme dans 55% des cas et la conformité globale était de 12,35% (Tableau 3).

**Tableau 3:** répartition selon les conformités

Conformité	Effectif	Proportion (%)
**Indication de l'antibiotique**		
Oui	188	55, 3
Non	152	45,7
**Molécule**		
Oui	135	39,7
Non	205	60,3
**Posologie**		
Oui	110	32,35
Non	230	67,65
**Voie d'admission**		
Oui	340	100
Non	0	0
**Durée**		
Oui	42	12,35
Non	298	87,65
**Réévaluation**		
Oui	108	31,76
Non	232	68,24
**Globale**		
Oui	42	12,35
Non	298	87,64
**Total**	**340**	**100**

**Les facteurs associés à la conformité globale:** en analyse multivariée, l'hospitalisation demeure un facteur de risque majeur (OR ajusté = 0,11 [0,03-0,42], p = 0,001), soit 89% de réduction de la probabilité de conformité (Tableau 4).

**Tableau 4:** analyses univariée et multivariée des facteurs associés à la conformité globale

Variables	n (%)	OR brut [IC95%]	p	ORa [IC95%]	p
**Âge (par année)**	135 (31,1%)	1,04 [1,00-1,08]	0,037	1,01 [0,96-1,06]	0,810
**NI (réf: NS)**					
Primaire	36 (33,3%)	4,00 [1,15-13,95]	0,030	2,52 [0,56-11,35]	0,229
Secondaire	44 (40,9%)	5,54 [1,67-18,40]	0,005	2,18 [0,48-9,88]	0,312
Supérieur	19 (42,1%)	5,82 [1,46-23,17]	0,013	1,39 [0,24-8,16]	0,713
**Résidence (réf: Urbain)**					
Rural	48 (10,4%)	0,16 [0,06-0,44]	<0,001	0,40 [0,12-1,36]	0,141
**Type de suivi (réf: Consultation)**					
Hospitalisation	69 (7,2%)	0,06 [0,02-0,17]	<0,001	0,11 [0,03-0,42]	0,001
Durée hospitalisation (jour)	69 (7,2%)	0,51 [0,27-0,94]	0,032	Non inclus	-
**Prescripteur (réf: MS)**					
Titulaire	47 (51,1%)	3,19 [1,46-6,96]	0,004	1,15 [0,42-3,16]	0,782

Réf: Référence; NI: niveau d'instruction; NS: non scolarisé; MS: médecin en spécialisation OR: Odds Ratio; ORa: Odds Ratio ajusté; IC95%: Intervalle de confiance à 5%

## Discussion

### Limites de l'étude

Notre travail constituait un audit rétrospectif. Ce type d'étude présente des atouts, notamment celui de limiter certains biais fréquemment rencontrés dans les études prospectives, tels que le biais d'information ou d'observation, ainsi que l'effet *Hawthorne*. Le biais d'information/observation correspond à des erreurs systématiques dans la collecte des données, tandis que l'effet *Hawthorne* traduit une modification du comportement des participants liée à la conscience d'être observés. Toutefois, certaines limites doivent être soulignées: la qualité de l'archivage, l'usage de dossiers manuscrits et l'exclusion de dossiers incomplets, susceptibles d'avoir introduit un biais de sélection et de limiter la représentativité des résultats.

### Conformité de l'indication

Dans notre étude, près de la moitié des prescriptions (45,7%) n'étaient pas conformes aux recommandations, un taux comparable à celui rapporté par Fortes *et al*. à Dakar (46%) [13], mais supérieur à ceux décrits en Côte d'Ivoire (24%) [7], en Tunisie (26 %) [5] et en France (7%) [4]. Plusieurs pathologies non infectieuses, telles que les grossesses extra-utérines, le paludisme grave ou l'anémie, ont bénéficié de prescriptions antibiotiques injustifiées. Ces écarts pourraient être liés au contexte local marqué par un environnement sanitaire perçu comme à haut risque infectieux (promiscuité, coupures d'eau, vétusté des équipements), conduisant à des prescriptions « préventives » excessives. Par ailleurs, le guide pratique de bonne prescription des antibiotiques recommande l'orientation des prescriptions par l'antibiogramme. Cependant le faible recours aux examens complémentaires (NFS: 42,6%; examens bactériologiques: 3,8%) limite la capacité à affiner le diagnostic et favorise l'usage inapproprié des antibiotiques.

### Conformité du choix de la molécule

Les bêta-lactamines, dominées par la ceftriaxone (24,4%), étaient les plus prescrites, rejoignant les observations de plusieurs études africaines [6,13]. Toutefois, la non-conformité atteignait 28,2%, proche des taux rapportés à Angers (17%) [14] ou à Nancy (22 %) [15], mais inférieure à celui observé à Dakar (46%) [13]. L'insuffisance de diffusion du guide (référentiel national) et la non-disponibilité de certaines molécules recommandées (ex. céfazoline pour l'antibioprophylaxie chirurgicale) pourraient expliquer ces résultats. Cela met en évidence la nécessité d'une stratégie de diffusion active du guide, intégrant sensibilisation, formations ciblées et adaptation des protocoles aux réalités locales.

### Conformité de la posologie et de la durée

La posologie n'était conforme que dans 32,3% des cas. Ce constat rejoint les travaux de Rémy *et al*. [15], mais contraste avec les résultats tunisiens plus favorables [5]. L'absence fréquente de la mesure du poids entrave le calcul précis des doses, exposant les patientes à des sous- ou surdosages, sources d'échec thérapeutique et de résistances. Concernant la durée, 87,6% des prescriptions étaient non conformes, un taux plus élevé que dans certaines études européennes (22-28 %) [14,15]. Dans notre contexte, la prescription par boîtes en consultation et l'absence de relais oral après hospitalisation ont conduit à des durées trop courtes. La rationalisation des durées constitue donc un levier majeur d'amélioration.

### Conformité globale

La conformité globale aux recommandations du guide n'était que de 12,4%. Bien que difficilement comparable aux données de la littérature en raison de méthodologies hétérogènes, ce taux est nettement inférieur à ceux rapportés à Rouen (47%) [4] ou à Dakar (54 %) [13]. Après l'analyse multivariée, l'hospitalisation s'est révélée comme le seul facteur associé à une prescription inappropriée des antibiotiques (OR ajusté = 0,11 [0,03-0,42], p = 0,001), soit 89% de réduction de la probabilité de conformité. Ces écarts traduisent une faible appropriation du guide national par les prescripteurs, particulièrement par les médecins en formation qui constituent la majorité des prescripteurs dans le service. Ce phénomène s'explique par plusieurs déterminants interdépendants. En premier lieu, l'insuffisance de formation continue et de supervision par les médecins titulaires limite la mise en pratique des recommandations. Ensuite, la complexité des cas pris en charge en milieu hospitalier, où la sévérité des pathologies et l'absence fréquente de confirmation microbiologique favorisent un recours empirique à des molécules à large spectre. Enfin des contraintes structurelles, telles que l'insuffisance des examens paracliniques et les ruptures d'approvisionnement, peuvent contraindre aux choix thérapeutiques non conformes. Cette analyse confirme la nécessité impérative d'une stratégie multidimensionnelle et intégrée. Celle-ci doit combiner une diffusion active et des formations continues ciblées sur le guide, un renforcement de la supervision clinique, et la mise en place systématique d'audits avec rétro-information pour les prescripteurs. En parallèle, il est crucial d'agir sur le système en améliorant les capacités diagnostiques (notamment microbiologiques), en garantissant la disponibilité des médicaments recommandés. Seule une telle approche permettra d'inverser la tendance et de promouvoir un usage rationnel des antibiotiques.

## Conclusion

Le bon usage des antibiotiques demeure un enjeu majeur de santé publique, particulièrement dans les pays à ressources limitées comme le Burkina Faso. Malgré l'élaboration d'un guide national, notre étude montre un écart important entre les pratiques de prescription et les recommandations officielles au DGOMR du CHUSS. Les principales insuffisances concernaient l'indication, le choix de la molécule, la posologie et surtout la durée du traitement. Ce travail a constitué la première évaluation de la mise en œuvre du guide au niveau hospitalier et met en évidence des pistes d'amélioration concrètes.

### Etat des connaissances sur le sujet

- Les antibiotiques représentent l'une des classes médicamenteuses les plus prescrites en milieu hospitalier ;
- Le mauvais usage des antibiotiques favorise l'émergence de résistances antimicrobiennes, enjeu majeur de santé publique à l'échelle mondiale;
- Le Burkina Faso a élaboré un guide pratique pour la bonne prescription des antibiotiques en 2019 afin de promouvoir leur usage rationnel.

### Contribution de notre étude à la connaissance

- Première évaluation de l'usage du guide pratique pour la bonne prescription des antibiotiques au Burkina Faso ;
- Important écart entre les pratiques de prescription et les recommandations du guide.
- Nécessité d'une diffusion active et de formations continues sur le guide pratique pour la bonne prescription des antibiotiques au Burkina Faso.

## Remerciements

Les auteurs remercient chaleureusement l'ensemble du personnel médical et paramédical du Centre Hospitalier Universitaire Souro Sanou pour leur collaboration et leur disponibilité tout au long de la collecte des données. Nous exprimons également notre gratitude aux responsables administratifs qui ont autorisé la réalisation de cette étude.
